# MicroRNAs in the Diagnosis of Malignancy of Supratentorial Brain Gliomas and Prognosis of Disease Progression

**DOI:** 10.7759/cureus.35906

**Published:** 2023-03-08

**Authors:** Evgeny V Stupak, Yulia A Veryasikina, Sergey E Titov, Arsen S Askandaryan, James C Hiana, Igor F Zhimulyov, Vyacheslav V Stupak

**Affiliations:** 1 Department of Neurosurgery, Novosibirsk Research Institute of Traumatology and Orthopedics n.a. Ya.L. Tsivyan, Novosibirsk, RUS; 2 Laboratory of Genetics, Institute of Molecular and Cell Biology, Siberian Branch of the Russian Academy of Sciences, Novosibirsk, RUS; 3 Genetics, AO Vector-Best, Novosibirsk, RUS; 4 Department of Psychiatry, Jamaica Hospital Medical Center, New York City, USA; 5 Department of Neurology, State University of New York Downstate Medical Center, New York City, USA

**Keywords:** predictor of survival, molecular genetic study, real-time pcr, microrna expression, microrna, gliomas of different malignancy, brain gliomas

## Abstract

Introduction: The study of brain tumors has shown that microRNAs can act as both oncogenes and tumor suppressors and, consequently, can be used as biomarkers for the diagnosis and prognosis of such tumors. Thus, big interest arises in the role of microRNA and its part in oncogenesis in the human brain to find key molecules that can act as tumor markers for diagnostic and prognostic purposes, as well as potential therapeutic agents.

Study aim: The sim of this study was to assess histological, molecular, and genetic metrics in patients with supratentorial gliomas, and indicate diagnostic and prognostic abilities of microRNA usage as biomarkers of the grade of malignancy of the tumor.

Materials and methods: Clinical and genetic studies were performed in 107 operated patients with supratentorial gliomas of different malignancies. The expression levels of 10 microRNAs (-16, -21¸ -31, -124, - 125b, -181b, -191, -221, -223, and -451) were analyzed using real-time polymerase chain reaction (PCR). The results were analyzed statistically using Statistica 12.0 (Statistica, Hamburg, Germany) and GraphPad Prism 9 software (GraphPad Software Inc., Boston, Massachusetts, United States).

Results: Based on a comprehensive statistical analysis involving the database of the clinical results of treatment of all 107 patients (combined treatment methods, quality of life, and survival) and microRNA expression levels, specific profiles of microRNA expression typical of different histotypes of gliomas of different malignancy were identified, the prognostic significance of the studied microRNAs as potential predictors of survival in patients with brain gliomas was determined, and microRNAs with the highest prognostic value were identified among them.

## Introduction

Glioma is aggressive and the most widespread primary CNS tumor. Its prevalence varies from 13 to 18 cases per 100,000 people per year. More than half of gliomas are malignant. Median survival is 10-14 months [1].

There are many new technologies involved in the treatment of brain gliomas that get better every year. Neuronavigational control, neurophysiological monitoring, and intraoperative fluorescence are currently used during cytoreductive tumor excision. During the paratumarous excision of the tumor node, some cancer cells remain and cause continued tumor growth. There are chemotherapy and radiation therapy options besides surgical treatment. Despite the multidisciplinary approach to treatment, stabilization of the process is rarely feasible [2].

Besides developing and improving treatment methods for patients with high-grade gliomas, the prognostic possibility of this pathology is an actual problem. Until recently, an indication of adjuvant therapy depended on the grade of malignancy based on the 2007 WHO classification and the prognosis of the brain glioma patients [3]. With the development of molecular-genetic studies in the field of CNS tumors, new factors responsible for indications for chemo and radiation therapy appeared. Some of them are O(6)-methylguanine-DNA methyltransferase (MGMT) status [4], which assesses somatic mutations in *IDH1 *and *IDH2* genes, 1p/19q co-deletion and many others which are much better correlated with life expectancy. The new 2016 and 2021 [5,6] histologic classification also indicates it. Despite the multidisciplinary approach, median survival stays short, and tumor recurs, leading to patients' deaths.

MicroRNA (miRNA) can be used as one of the molecular markers of prognosis of the brian gliomas course. miRNA is a non-coding RNA that regulates posttranscriptional gene expression. It causes deadenylation and degradation of mRNA targets in various biological processes like proliferation, differentiation, invasion, and apoptosis [7]. Some data indicate that different miRNAs can regulate hundreds of mRNA, showing that miRNA participates in the tumor processes [8]. Each year the number of publications about the role of miRNA in CNS gliomas increases. Research has shown that miRNA can function as oncogenes or suppressor genes in gliomas [8]. This causes great interest in researching miRNA's effect on oncogenesis processes and the discovery of the spectrum of crucial molecules, which potentially could be predictors of the prognosis of the disease course and survival of the patients with this disease [8,9].

The study aims to assess histological, molecular, and genetic metrics in patients with supratentorial gliomas and indicate diagnostic and prognostic abilities of miRNA usage as biomarkers of the grade of malignancy of the tumor.

## Materials and methods

Clinical and genetic studies were carried out in 107 operated patients with supratentorial gliomas of different levels of malignancy at Novosibirsk Research Institute of Traumatology and Orthopedics n.a. Ya.L. Tsivyan of the Ministry of Health of the Russian Federation. A total of 1 cm^3^ of biopsies of the tumor and normal brain tissue were taken at a distance of a minimum of 2 cm from the neoplasm during encephalotomy when accessing the tumor for its resection using neuronavigation. RNAlater solution was used to stabilize RNA during the storage and transportation of surgical samples, which allows for avoiding sample freezing in liquid nitrogen for RNA isolation from cells and tissues. The mean patient age was 48.8 ± 14 years. It ranged from six to 83 years. Among the operated patients, there were 39 (36.4 %) individuals ≤ 48 years and 68 (63.6 %) operated patients ˃ 48 years. There were 62 (57.9 %) males and 45 (42.1 %) females.

A total of 107 operated patients (91 primary and 16 secondary gliomas) were divided into three groups depending on glioma malignancy: 17 (16 %) individuals with grade II gliomas (group 1), 30 (28 %) cases with grade III gliomas (group 2), and 60 (56 %) patients with grade IV gliomas. Supratentorial gliomas were histologically classified according to the histopathological classification adopted by the WHO in 2016 [5].

The quality of patients’ life (QOL) was assessed using the Karnofsky performance status scale [10]. According to this scale, the patients' functional state was 70 ± 1.0 points before surgery.

Neoplasms were resected totally in 67 (62.6 %) cases and subtotal in 29 (27.1 %) individuals, while puncture biopsy was performed in 11 (10.3 %) patients using neuronavigation. The radicality of surgical treatment in patients was assessed by contrast-enhanced CT imaging the next day after surgery. Preference was given to contrast-enhanced MRI; in cases when CT was performed before surgery, contrast-enhanced CT was performed after surgery for comparison.

Patients with glioblastoma diagnosis after the operation received adjuvant therapy chemo- and radiotherapy 56 (52%). Ten patients (9%) who received radiotherapy with 60 Hz were diagnosed with grade III and grade IV gliomas. Six (5%) patients with grade III gliomas received Temodal chemotherapy. The remaining patients, 35 (34%), did not receive additional treatment after the operation. The reason for refusing adjuvant therapy in the postoperative period was a severe sub-compensated state: the median QOL value was 58 Karnofsky points.

The median survival of patients with grade II-IV supratentorial gliomas was 15 months after surgery and histological diagnosis. The median survival was 48 months in individuals with grade II tumors, 43 months in cases with grade III gliomas, and eight months in patients with grade IV tumors. The average life expectancy was 43, 42, and 10 months in individuals with grade II, III, and IV gliomas, respectively.

The expression levels of 10 miRNAs (-16, -21¸-31, -124, - 125b, -181b, -191, -221, -223, and -451) were measured by real-time polymerase chain reaction (PCR) on a CFX96 amplifier (Bio-Rad Laboratories, Inc., Hercules, California, United States) [11]. Total RNA was isolated using a RealBest extraction 100 kit (AO Vector-Best, Novosibirsk, Russia). Reverse transcription (RT) was performed using a RealBest Master Mix RT reaction mixture (AO Vector-Best, Novosibirsk, Russia). Small RNA U58 was used as a reference gene.

Statistical data analysis was carried out using the non-parametric Mann-Whitney U test. A decision tree was generated using the classification and regression tree (C&RT) algorithm, the multivariate Cox regression analysis, and the receiver operating characteristic (ROC) analysis in Statistica 12.0 (Statistica, Hamburg, Germany) and GraphPad Prism 9 software (GraphPad Software Inc., Boston, Massachusetts, United States).

## Results

Expression levels of 10 miRNAs (-16, -21¸ -31, -124, -125b, -181b, -191, -221, -223, and -451) were assessed in tumor samples and adjacent normal brain tissues collected intraoperatively from 107 patients. At the beginning of our research, we analyzed and compared the results of all glioma tissue samples with morphologically unchanged brain tissue of the same patient. We received statistically significant results for miRNA -21, -124, and -223 (p < 0,05) (Table 1).

**Table 1:** Median microRNA expression levels in all glioma sample pairs of different malignancies and corresponding samples of adjacent normal brain tissue Note: p < 0.05 – differences in miRNA expression levels assessed using the Mann–Whitney U test are statistically significant; these differences are highlighted in bold. AUC: area under the curve; NBT: normal brain tissue

miRNA	Tumor/Normal brain tissue (107/107)		
	Tumor/(NBT)	P-value	AUC
microRNA -16	1.27	0.178294	0.5607
microRNA -21	2.51	0.000411	0.659
microRNA -31	0.58	0.105235	0.5719
microRNA -124	0.54	0.044237	0.5912
microRNA -125b	1.85	0.429326	0.5354
microRNA -181b	1.22	0.665813	0.519
microRNA -191	0.79	0.809246	0.5107
microRNA -221	0.85	0.338156	0.5424
microRNA -223	2.58	0.013701	0.6083
microRNA -451	0.64	0.972783	0.5019

The expression levels of 10 miRNAs were evaluated depending on glioma malignancy and compared to the parameters of normal brain tissues (Table 2).

**Table 2:** Comparison of median microRNA levels in tumor samples and adjacent normal brain tissue p < 0.05 – differences in microRNA expression levels assessed using the Mann–Whitney U test are statistically significant; these differences are highlighted in bold. NBT: normal brain tissue; AUC: area under the curve

microRNA	Grade II (n=17)			Grade III (n=30)			Grade IV (n=60)		
	Tumor/(NBT)	P-value	AUC	Tumor/(NBT)	P-value	AUC	Tumor/NBT	P-value	AUC
microRNA -16	1.54	0.722	0.54	0.73	0.717	0.528	1.43	0.170	0.593
microRNA -21	0.67	0.782	0.52	1.73	0.244	0.588	5.58	0.0006	0.733
microRNA -31	1.35	0.821	0.51	0.38	0.023	0.672	0.46	0.319	0.566
microRNA -124	0.88	0.968	0.50	0.55	0.214	0.598	0.24	0.028	0.647
microRNA -125b	0.99	0.597	0.55	0.63	0.266	0.584	3.78	0.052	0.630
microRNA -181b	1.56	0.969	0.51	0.85	0.615	0.538	1.64	0.487	0.546
microRNA -191	0.64	0.734	0.53	0.63	0.440	0.558	1.02	0.712	0.524
microRNA -221	0.72	0.597	0.55	0.25	0.035	0.659	1.87	0.787	0.518
microRNA -223	1.71	0.651	0.54	0.50	0.515	0.549	70.87	0.0001	0.760
microRNA -451	0.70	0.843	0.51	1.10	0.589	0.541	0.20	0.307	0.570

A statistically significant (p < 0.05) decreased expression levels of miRNAs -31 and -221 were noted in the group of supratentorial gliomas of grade III anaplasia consisting of 30 patients (Table 2). Analysis of the largest group with glioblastomas (grade IV), which included 60 patients, showed a statistically significant increase in expression levels of miRNAs -21 and -223 in tumor tissue compared to normal brain tissue (p < 0.05). A statistically significant increase in -21, -223 miRNA expression was discovered (p<0.05). The expression levels of miRNAs -21 and -223 were greater than five-fold and 70-fold higher in the tumor compared to the normal brain. A significant decrease in the level of miRNA-124 level (p < 0.05) was also noted in glioblastoma tissues (Table 2).

**Figure 1:**
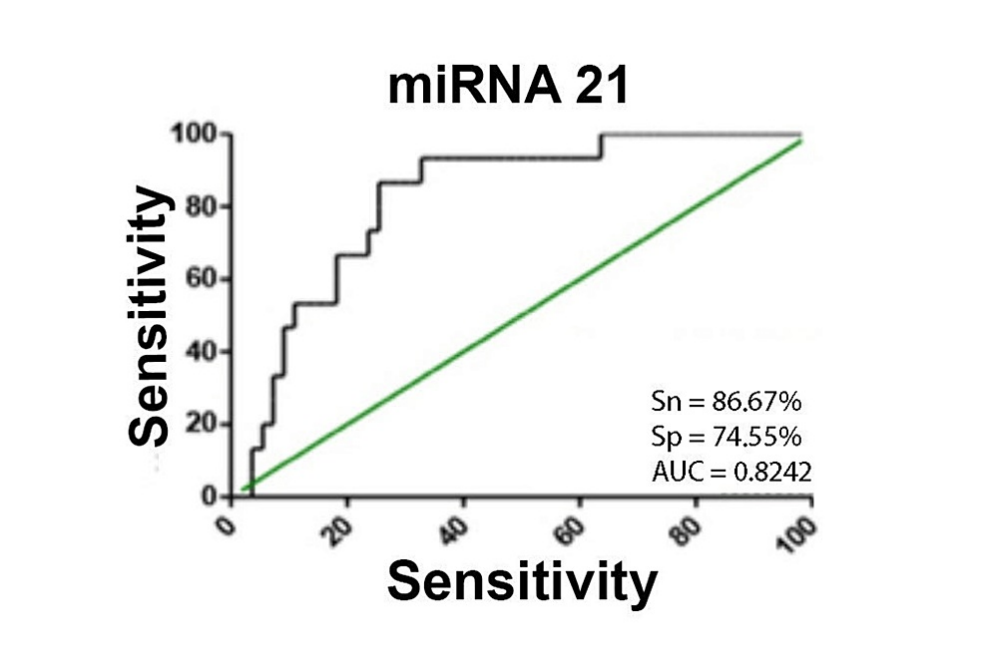
ROC analysis: a – microRNA-21 (grade II/grade IV). miRNA: microRNA; ROC: receiver operating characteristic; AUC: area under the curve; Sn: sensitivity; Sp: specificity

Next, we compared miRNA expression levels between gliomas of various degrees of malignancy. Analysis was performed between grade II and III, II and IV, and grade III and IV gliomas. The expression level of miRNA-21 was increased statistically significantly seven-fold in grade IV gliomas compared to grade II gliomas (Table 3). The area under the curve (AUC) values for miRNA-21 are in the range of 0.8-0.9 on the ROC curve, which indicates the excellent significance of these criteria in relation to the differences between tumors of different malignancies (Figure 1).

**Table 3:** Comparison of median microRNA expression levels among glioma tissue samples of different malignancies (grades II-IV). p < 0.05 – differences in microRNA expression levels assessed using the Mann–Whitney U test are statistically significant; these differences are highlighted in bold. AUC: area under the curve

microRNA	Grade II/Grade III			Grade II/Grade IV			Grade III/Grade IV		
	Grade II/Grade III	P-value	AUC	Grade II/Grade IV	P-value	AUC	Grade III/Grade IV	P-value	AUC
microRNA -16	0.86	0.882	0.514	1.90	0.178	0.615	1.62	0.138	0.594
microRNA -21	2.70	0.264	0.6	6.94	0.00013	0.824	18.75	0.00016	0.745
microRNA -31	0.42	0.487	0.562	2.95	0.505	0.554	1.25	0.063	0.537
microRNA -124	0.73	0.501	0.561	1.47	0.787	0.523	1.07	0.212	0.520
microRNA -125b	2.34	0.933	0.508	0.74	0.841	0.517	1.73	0.668	0.538
microRNA -181b	1.17	0.262	0.598	1.24	0.125	0.626	1.44	0.716	0.532
microRNA -191	1.59	0.690	0.535	1.43	0.257	0.593	2.28	0.077	0.572
microRNA -221	1.09	0.860	0.511	2.37	0.164	0.613	2.58	0.019	0.657
microRNA -223	2.15	0.126	0.642	1.72	0.126	0.628	3.69	0.909	0.602
microRNA -451	0.57	0.430	0.569	2.67	0.647	0.542	1.52	0.079	0.534

Comparison of miRNA expression between grade III and IV gliomas revealed significant differences in miRNA-21 and -221 levels; their expression levels in glioblastoma tissues were increased by 18.7- and 2.5-fold, respectively, compared to grade III neoplasms (Table 3).

To determine the accuracy of the analysis of glioma malignancy of 10 miRNAs at once, a decision tree was generated using the C&RT algorithm. As a result, six miRNAs were included in the decision tree: -21, -221, -223, -125b, -191, and -124. For grade II tumors, the diagnostic values were the following: 90% specificity, 76.5% sensitivity, 87.8% overall accuracy, and 83.2 AUC. For grade III, the following values were obtained: 93.4% specificity, 74.2% sensitivity, 87.8% overall accuracy, and 83.8 AUC. For grade IV, the values were 89.6% specificity, 88.1% sensitivity, 87.8% overall accuracy, and 88.8 AUC.

We have used Cox multifactor regression analysis to assess the possibility of using miRNA as a potential predictor for the survival of patients with various grades of gliomas. Simultaneous effects on the survival rate of miRNA (potential prognostic markers), age, type of adjuvant therapy, and the grade of malignancy were analyzed. All metrics are shown in Table 4.

**Table 4:** Cox regression analysis in patients with glioma of various degrees of malignancy by 10 microRNA expression levels, age, type of adjuvant therapy, and malignancy of the tumor. Significant parameters (p < 0.05) are highlighted in bold.

Factor		Parameter value	P-value	Odds ratio
microRNA	microRNA -16 ↑	0.057	0.625	1.06
microRNA	microRNA -21 ↑	0.206	0.003	1.23
microRNA	microRNA -31 ↑	0.199	0.0335	1.22
microRNA	microRNA -124 ↑	0.014	0.805	1.01
microRNA	microRNA -125b ↑	0.027	0.801	1.03
microRNA	microRNA 181b ↓	-0.084	0.332	0.92
microRNA	microRNA -191 ↓	-0.170	0.298	0.84
microRNA	microRNA 221 ↓	-0.206	0.022	0.82
microRNA	microRNA -223 ↑	0.101	0.046	1.11
microRNA	microRNA -451a ↓	-0.007	0.863	0.99
Chemotherapy	No chemotherapy	0.450	0.014	2.46
Radiation therapy	No radiation therapy	0.375	0.047	2.12
Grade II– Grade IV	Grade II	-2.094	0.000017	0.01
Age ≤ 48/˃ 48 years	Age ≤ 48 years	-0.618	0.0001	0.29

**Figure 2:**
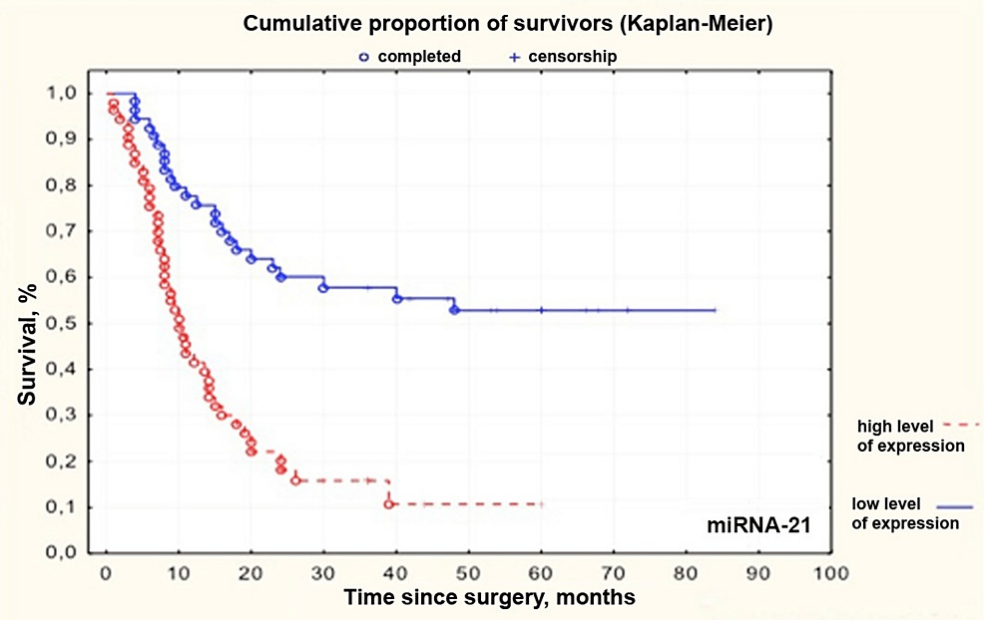
Kaplan–Meier curves for patients with high and low levels of microRNA-21 in grade II-IV gliomas miRNA: microRNA

**Figure 3:**
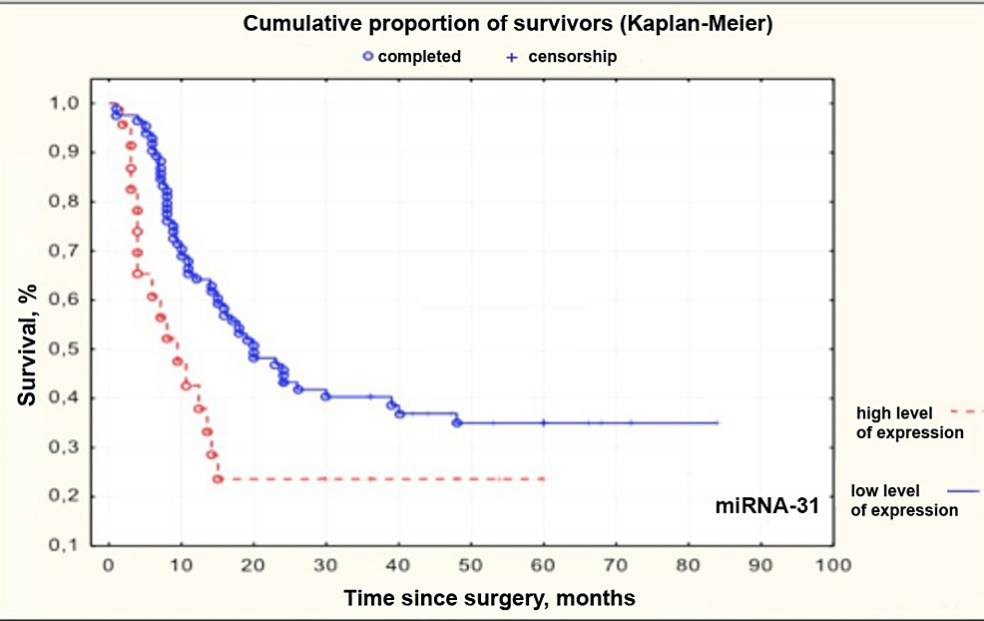
Kaplan–Meier curves for patients with high and low levels of microRNA-31 in grade II-IV gliomas miRNA: microRNA

**Figure 4:**
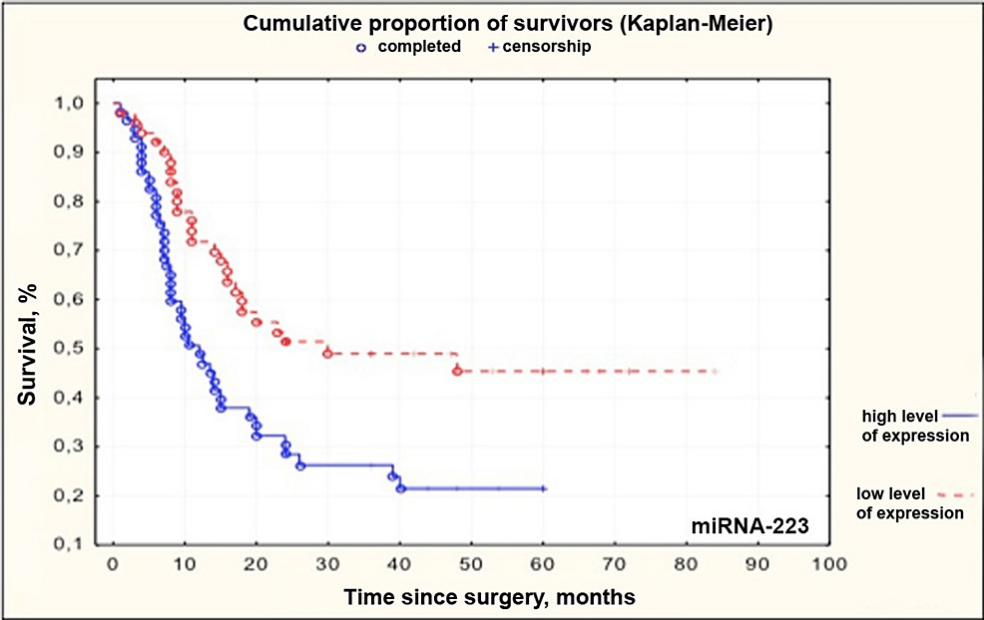
Kaplan–Meier curves for patients with high and low levels of microRNA-223 in grade II-IV gliomas miRNA: microRNA

**Figure 5:**
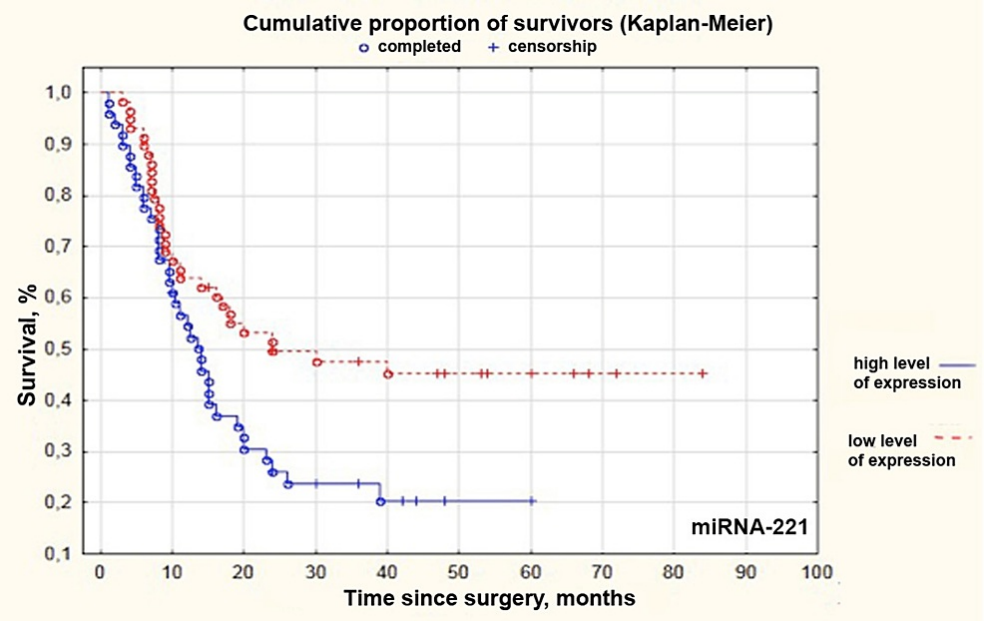
Kaplan–Meier curves for patients with high and low levels of microRNA-221 in grade II-IV gliomas miRNA: microRNA

MiRNAs -21, -31, -223, and -221 had the highest correlation with patient survival of all the studied miRNAs. We compared groups with high and low levels of miRNAs -21, -31, -223, and -221. ROC curve was generated for each miRNA. An assessment of threshold values of relative expression levels was made. This level corresponded to 4.443, -0.09804, 1.315, and -0.531 for miRNAs -21, -31, -223, and -221, respectively. After group comparison, Kaplan-Meier curves were obtained (Figures 2-5).

Median survival in groups with low and high levels of miRNAs -21, -31, -223, and -221 and patients undergoing adjuvant therapy depending on age is presented in Table 5.

**Table 5:** Median survival in operated patients depending on the selected prognostic factors. Increased expression of all four microRNAs, particularly microRNA -21 (HR = 1.228; 95%CI 1.073-1.407; p < 0.0030), microRNA -31 (HR = 1.221; 95%CI 1.016-1.467; p < 0.0335), microRNA -223 (HR = 1.106; 95%CI 1.002-1.221; p < 0.0460) and microRNA -221 (HR = 0.814; 95%CI 0.683-0.970; p < 0.0216), lack of chemotherapy (HR = 2.461; 95%CI 1.199-5.050; p < 0.0141) and radiotherapy (HR = 2.116; 95%CI 1.011–4.430; p < 0.0467), and age older than 48 years old (HR = 0.291; 95%CI 0.154-0.547; p < 0.0001) significantly correlated with a decrease in patient survival. Me: median survival; HR: hazard ratio

Factor	Median survival	
microRNA -21	Low expression level (Me 24 months)	High expression level (Me 10 months)
microRNA -31	Low expression level (Me 19.5 months)	High expression level (Me 9 months)
microRNA-223	Low expression level (Me 24 months)	High expression level (Me 10.5 months)
microRNA-221	Low expression level (Me 24 months)	High expression level (Me 12.5 months)
Chemotherapy	Chemotherapy (Me 20 months)	No radiation therapy (Me 8 months)
Radiation therapy	Radiation therapy (Me 20 months)	No radiation therapy (Me 8.5 months)
Age ≤ 48/˃ 48 years	Age ≤ 48 years (Me 36 months)	Age ˃ 48 years (Me 10.2 months)

## Discussion

In this research, we received the results of the data analysis of the complex clinical treatment of 107 patients with gliomas of different grades of malignancy and different expression levels of 10 miRNAs. We defined the prognostic significance of miRNA as markers for assessing gliomas grade level and as potential survival predictors for the patients. Among them, we revealed the ones with the highest prognostic values.

We found significant differences in the level of five miRNAs between glioma tissue and externally unchanged medulla located at a distance of at least 2 cm from it: two oncogenic (-221 and -21) and two tumor suppressive (-31, -124, and -223) ones. These miRNAs can be used as markers in assessing the malignancy of these tumors. Analysis of expression levels of the studied miRNAs revealed their specific profiles typical of gliomas of different malignancies and the dependence of these parameters on their malignancy.

The diagnostic parameters for generating a decision tree to determine glioma malignancy based on measuring the expression levels of six miRNAs (-21, -124, -125b, -191, -221, and -223) are the following: 90,1% specificity, 76.6% sensitivity, 87.9% overall accuracy, and 83.3 AUC for grade II gliomas; 93.5% specificity, 74.1% sensitivity, 87.7% overall accuracy, and 83.9 AUC for grade III: gliomas; 89.7% specificity, 88% sensitivity, 87.7% overall accuracy, and 88.9 AUC for grade IV gliomas.

Modern research findings indicate that individual miRNAs can act as patient survival markers depending on their expression levels. For instance, Srinivasan et al. analyzed the expression levels of 10 miRNAs in a sample of 222 individuals diagnosed with brain glioblastoma [12]. The study revealed that seven miRNAs (-31, -222, -148a, -221, -146b, -200b, and -193a) were associated with an unfavorable prognosis of patient survival, and three miRNAs (-20a, -106a, and -17-5p) indicated good survival prognosis. Studies by Jiang et al. demonstrated a five-fold and a 32-fold increase in miRNA-182 levels in primary glioma and glioblastoma samples, respectively, compared to the tumor-adjacent tissue [13]. The level of circulating miRNA-182 was higher in these patients compared to healthy people (p < 0.001). Expression levels correlated with tumor malignancy. Based on this data, the authors concluded that miRNA-182 could be a promising biomarker for early diagnosis and survival prognosis in patients with the disease [13]. Lan et al. proved that a decrease in the expression of miRNA-144-3p is positively correlated with a low overall survival rate. It was discovered that not only levels of expression of miRNA-144-3p decreased in glioma tissues compared to the paratumoral, but especially in high-grade gliomas compared to low-grade gliomas. In addition, glioma patients with low miR-144-3p levels had poorer survival rates [14]. Ye et al. studied miRNA-183 levels and found that they were higher in tumors compared to paratumoral brain tissue and reached their maximum in high-grade gliomas [15]. Similar studies have shown that miRNA-183 expression levels significantly correlate with patient survival. Patients with high miRNA-183 levels had significantly shorter overall survival and progression-free survival than patients with low miRNA-183 expression. In addition, univariate and multivariate analysis showed that miRNA-183 level is an independent prognostic survival parameter for patients with these types of neoplasms [16].

Generated data from our study from molecular-genetic data of expression of 10 miRNAs in 107 patients with brain gliomas of different grades of malignancy was compared to the clinical data of the patients. It was shown that among 10 studied RNAs, high expression of certain miRNA (-31, -21, -223, and -221), lack of chemo- and radiotherapy, and age over 48 years old were positively correlated with the decreased survival rate of the patients, which indicated poor prognosis in the treatment of the disease. It allows us to use it as a potential survival predictor in patients with supratentorial brain gliomas.

MiRNA-21 is widely present in the human body. It is oncogenic, and its level is increased in the tumor tissues, including the tissues of gliomas [17]. Various data sources show that the miRNA expression level is increased 5-15 times compared to the normal tissue [16-18]. The oncogenic effect of miRNA-21 in human glioma cells is implemented through the suppression of such tumor suppressor genes as *TAp63*, *HNRPK*, *JMY*, *TOPORS*, *RECK*, *TP53BP2*, *TIMP3*, *PDCD4*, and *TGFBR2/3* [19]. Its overexpression leads to enhanced proliferation, invasion, and decreased apoptosis of tumor cells. In addition, a low level of this miRNA, according to the Cancer Genome Atlas (TCGA), is weakly associated with increased survival. Inhibition of miRNA-21 decreases estimated glomerular filtration rate (eGFR) expression and causes cell cycle arrest at the G1/S phase, resulting in tumor growth inhibition [20]. Wu et al. demonstrated that glioma patients with high miRNA-21 expression had worse parameter values, and operated individuals with a low level of this miRNA had higher survival rates [21]. The results presented in this work confirmed that increased miRNA-21 expression (hard ratio (HR) = 1.228; 95%CI 1.073-1.407; p < 0.0030) is associated with reduced survival of patients with cerebral gliomas.

MiRNA-221 is oncogenic and targets proteins p27 and p57, and *PTEN*, *TIMP3*, *PUMA*, and *Cx43* genes regulating the cell cycle and cell survival processes [22]. Its increased expression is observed in brain glioma tissues [23]. A statistically significant increase in the level of this miRNA in gliomas of high malignancy degree (grade III-IV) compared with normal brain tissues was noted in our study [18]. MiRNA -221 is oncogenic, it targets the p57 protein, and the *TIMP3* gene regulates the cell cycle and cell survival processes [22]. Its increased expression is observed in glioma tissues of the brain [23]. A statistically significant increase in the level of this miRNA in gliomas of high malignancy degree (grade III-IV) compared with normal brain tissues was noted in our study [18]. This work shows that increased miRNA-221 expression (HR = 0.814; 95%CI 0.683-0.970; p < 0.0216) significantly correlates with a decrease in the survival of operated patients.

MiRNA-31 acts as a tumor suppressor in CNS tumors. Its expression level correlates with tumor predisposition to invasion and metastasis. Our previous study noted a statistically significant decrease in this miRNA expression in grade III-IV gliomas compared to paratumoral tissue [18]. MiRNA -31 inhibits cell migration in glioma cell culture and indirectly regulates activation of the NF-kB transcription factor, angiogenesis, and E-cadherin level through the epithelial-to-mesenchymal transition [24]. In our study, increased miRNA-31 level significantly correlated with a sharp decrease in patient survival (HR = 1.221; 95%CI 1.016-1.467; p < 0.0335).

Studies by Huang et al. indicate that miRNA-223 can play different roles in different types of cancer and different tumor cell lines by targeting various functional genes [25]. Studies have shown that the miRNA-223 level in human glioblastoma cells U138, U373, U251, and A172 is higher than that in human fetal glial cells, thus indicating its increased expression in glioblastoma. Results obtained by Ding et al. demonstrate that its level is reduced in glioblastoma tissues [26]. The report shows that this miRNA can act as a tumor suppressor and a potential target in glioblastoma therapy. However, it remains unknown whether this miRNA is a tumor suppressor or an oncogene in glioblastoma [26]. Our data indicate increased expression of miRNA-223 in brain gliomas (HR = 1.106; 95%CI 1.002-1.221; p < 0.0460), which significantly correlates with a sharp decrease in patient survival after surgery.

MiRNA-124 is a tumor suppressor that is involved in neuronal differentiation. Its expression has been shown to be decreased not only in glioblastomas and oligodendrogliomas but also in medulloblastomas [27]. Our previous study and the study by Silber et al. have shown that the miRNA-124 level is statistically significantly reduced in gliomas, including glioblastomas, compared to the tumor-adjacent tissue [18,28]. This miRNA regulates the cell cycle at G0/G1 and inhibits the CDK6 kinase, which stimulates angiogenesis [19,28], leading to the formation of new vessels. The latter leads to further tumor growth and metastasis [29]. Transfection of glioma cells with miRNA-124 impedes cell migration [30].

In modern medical practice, a personalized approach plays a significant role in treating oncological pathology, including neurooncology. Discovered data of the last four miRNAs can be used in clinical practice to reveal the high-risk patients to provide adjuvant therapy in addition to the standard care protocol.

Limitations to our study include the low number of selected patients and differences between Grade II, III, and IV gliomas. Benign gliomas rarely cause general or focal neurologic symptoms. These patients seek care during the malignant progression. We plan to validate our results as the number of patients increases.

## Conclusions

During our research, we found miRNA profiles specific for gliomas of different malignancies. MiRNAs -21, -31, -124, -221, and -223 can be used as markers for assessing the malignancy of supratentorial gliomas. We built a decision tree to increase the precision of the malignancy grade assessment. This tree included miRNAs -21, -124, -125b, -191, -221, and -223. We also defined the prognostic significance of the studied miRNAs as potential survival predictors among glioma patients. We determined the highest prognostic significance among these miRNAs. Increased expression of miRNAs -21, -31, -223, and -221 in brain gliomas significantly correlates with a decrease in patient survival, indicates a poor disease prognosis, and can be used for predicting the survival of patients with supratentorial brain gliomas.
